# The Effects of smoking status and smoking history on patients with brain metastases from lung cancer

**DOI:** 10.1002/cam4.1058

**Published:** 2017-04-12

**Authors:** Rachel F. Shenker, Emory R. McTyre, Jimmy Ruiz, Kathryn E. Weaver, Christina Cramer, Natalie K. Alphonse‐Sullivan, Michael Farris, William J. Petty, Marcelo R. Bonomi, Kounosuke Watabe, Adrian W. Laxton, Stephen B. Tatter, Graham W. Warren, Michael D. Chan

**Affiliations:** ^1^ Department of Radiation OncologyWake Forest School of MedicineWinston‐Salem27157North Carolina; ^2^ Department of Medicine (Hematology & Oncology)Wake Forest School of MedicineWinston‐Salem27157North Carolina; ^3^ Department of Social Sciences and Health PolicyWake Forest School of MedicineWinston‐Salem27157North Carolina; ^4^ Department of Cancer BiologyWake Forest School of MedicineWinston‐Salem27157North Carolina; ^5^ Department of NeurosurgeryWake Forest School of MedicineWinston‐Salem27157North Carolina; ^6^ Department of Radiation OncologyMedical College of South CarolinaCharleston29425South Carolina; ^7^ Department of Cell and Molecular PharmacologyMUSCCharleston29425South Carolina

**Keywords:** Brain metastases, brain metastasis velocity, lung cancer, smoking cessation, stereotactic radiosurgery

## Abstract

There is limited data on the effects of smoking on lung cancer patients with brain metastases. This single institution retrospective study of patients with brain metastases from lung cancer who received stereotactic radiosurgery assessed whether smoking history is associated with overall survival, local control, rate of new brain metastases (brain metastasis velocity), and likelihood of neurologic death after brain metastases. Patients were stratified by adenocarcinoma versus nonadenocarcinoma histologies. Kaplan–Meier analysis was performed for survival endpoints. Competing risk analysis was performed for neurologic death analysis to account for risk of nonneurologic death. Separate linear regression and multivariate analyses were performed to estimate the brain metastasis velocity. Of 366 patients included in the analysis, the median age was 63, 54% were male and, 60% were diagnosed with adenocarcinoma. Current smoking was reported by 37% and 91% had a smoking history. Current smoking status and pack‐year history of smoking had no effect on overall survival. There was a trend for an increased risk of neurologic death in nonadenocarcinoma patients who continued to smoke (14%, 35%, and 46% at 6/12/24 months) compared with patients who did not smoke (12%, 23%, and 30%, *P* = 0.053). Cumulative pack years smoking was associated with an increase in neurologic death for nonadenocarcinoma patients (HR = 1.01, CI: 1.00–1.02, *P* = 0.046). Increased pack‐year history increased brain metastasis velocity in multivariate analysis for overall patients (*P* = 0.026). Current smokers with nonadenocarcinoma lung cancers had a trend toward greater neurologic death than nonsmokers. Cumulative pack years smoking is associated with a greater brain metastasis velocity.

## Introduction

The 2014 Surgeon General's Report concluded that smoking by cancer patients and survivors caused adverse outcomes through increased overall mortality, cancer‐specific mortality, risk for second primary cancer, and associations with increased cancer treatment toxicity 1. Smoking cessation has been linked to improved outcomes in lung cancer patients 2, 3, 4. Most of these improvements are thought to be related to decreased risk of a second primary lung cancers 5, improved tolerance of curative therapies 2, 3, 6, and general improvement of health status 4, 7. Smoking and tobacco products have been shown to alter biologic cancer pathways leading to increased proliferation, angiogenesis, migration, invasion, and decreased response to cytotoxic therapy 8, 9, suggesting that smoking cessation may remove a physiologic driver of cancer progression 10, 11.

A recent meta‐analysis demonstrated that mortality was decreased by 66% in early stage lung cancer patients who stopped smoking 9 and current smoking lung cancer patients enrolled in an opt‐out smoking cessation program had a 44% reduction in overall mortality after adjustment for age, stage, and performance status 8. However, there is little data on whether smoking or cessation affects outcomes in lung cancer patients with brain metastases.

The population of lung cancer patients with brain metastases is of interest because this is a population for which significant improvements in survival has occurred over the past two decades. These improvements have occurred particularly due to improvement in brain‐directed therapies 12 and improvement in control of extracranial disease 13. In addition, lung cancer patients represent just over one half of the approximately 170,000 patients in the US each year with brain metastases 14.

Oncology providers may be less likely to emphasize smoking cessation in metastatic patients 15 because these patients are thought to be incurable with short life expectancies. Metastatic patients have also not been included in many studies examining outcomes of smoking cessation 9. It is not known if smoking cessation affects outcomes in the context of lung cancer patients with brain metastases. The purpose of this study was to evaluate the relationship between smoking and outcomes in lung cancer patients with brain metastases.

## Materials and Methods

### Study population

Study patients had a diagnosis of nonsmall cell lung cancer and brain metastases treated with Gamma Knife stereotactic radiosurgery (SRS) between January 2000 and December 2013. Patients who had prior whole brain radiation therapy (WBRT) were excluded because WBRT was thought to affect clinical outcomes. Patient data in this database were entered prospectively after July 2008 as part of a broader study on outcomes following Gamma Knife SRS. This study was approved by the Wake Forest School of Medicine Institutional Review Board.

Patient electronic medical records were used to obtain patient socio‐demographic and cancer characteristics. Socio‐demographic factors included gender, age, performance status, smoking status, smoking history. Cancer characteristics included histology status of extracranial disease at time of SRS (progressive or stable), extent of extracranial disease at time of SRS (none, oligometastatic, widespread), number of brain metastases and disease‐specific Graded Prognostic Assessment (ds‐GPA). Extent of extracranial disease 16 and ds‐GPA 17 were defined as in prior reports.

### Radiosurgical technique

Radiosurgery was performed on the Leksell Gamma Knife Model B (years 2000–2004), C (2004–2009), and Perfexion (2009–2013) (Elekta AB, Stockholm). High‐resolution contrasted MRI was performed just prior to SRS. Median dose delivered to the tumor margin was 20 Gy (range: 10–24 Gy). Dose was generally prescribed to the 50% isodose line. Dose was prescribed according to guidelines published by Shaw et al. 18.

### Response assessment and follow‐up

Patient survival from time of SRS was determined from the electronic medical records and the Social Security Death Index. A local failure was determined as either: (1) pathologically proven from surgical specimen, or (2) included an increase in 25% of the volume of a lesion with a corresponding increase in perfusion on perfusion weighted MRI, as previously reported 13.

The brain metastasis velocity was calculated as previously reported by Farris et al. 19. In summary, the total number of new brain metastases over the course of a patient's lifetime were calculated and divided by the total time to last follow‐up MRI from time of original SRS. Brain metastasis velocity was calculated for each patient in the study to serve as a surrogate for the aggressiveness of reseeding the brain with new metastases.

Neurologic death was defined as previously reported by Lucas et al. 20. In brief, patients were considered to have neurologic death if they had progressive neurologic decline at the time of death. Also, patients with severe neurologic dysfunction who died of intercurrent disease were considered to have died from neurologic death.

### Smoking status of patients

Smoking status of patients was determined through the electronic medical records at the time of diagnosis of brain metastasis and thereafter. A patient was classified as a current smoker if they had evidence of active smoking or had <1 year of smoking cessation as patients who report less than 1 year of smoking cessation have been shown to be less reliable with continued cessation 21. Cumulative pack years of smoking were also determined through the medical records. Pack years were calculated by multiplying the number of packs of cigarettes smoked per day by the number of years the person has smoked. Never smokers were considered to have 0 pack years.

### Statistical analyses

Patient characteristics were stratified by adenocarcinoma versus nonadenocarcinoma histology due to the fact that smoking has been reported to be less of associated with adenocarcinoma than other histologies 22, 23 and because of the previously reported differences in natural history of brain metastases in these populations 16, 24. Stratified variables were then compared using chi‐squared tests for categorical variables and either a t‐test or Mann–Whitney U test for continuous variables. Median follow‐up and time‐to‐event outcomes were defined as the time from SRS to the time of most recent follow‐up or to the event of interest. Time‐to‐event outcomes were summarized using the Kaplan–Meier estimator with log‐rank tests performed for stratified outcomes. Cumulative incidences were estimated for neurologic death and local failure. Competing risks models were developed to estimate the single variable subdistribution hazard ratios (HR) associated with each predictor for each of these events. Linear regression was performed for predictor variables of interest for the outcome of new brain metastases over time, or brain metastasis velocity (BMV). For patients with a single distant brain failure (DBF) event, BMV was defined as the number of new metastases over the time from initial GKRS until the DBF event. For patients with multiple DBF events, BMV was estimated by performing a separate linear regression versus time for each patient to obtain the slope representative of the best fit line. Stepwise regression was used to identify the multiple regression model with the lowest AIC 25. Statistical analysis was performed using R version 3.2.1 software (R Foundation for Statistical Computing, Vienna, Austria).

## Results

### Patient population

Of 366 patients included in the analysis, the median age was 63, 54% were male and, 60% were diagnosed with adenocarcinoma. Current smoking was reported by 37% and 91% had a smoking history. Patient characteristics are summarized in Table 1. Median overall survival for the cohort was 8.0 (CI: 6.7–9.3) months. Median overall survival for adenocarcinoma and nonadenocarcinoma was 8.5 (CI: 7.2–10.8) and 6.9 (CI 5.4–8.9) months, respectively.

**Table 1:** Clinical and smoking characteristics of lung cancer patients with brain metastases receiving intracranial stereotactic radiosurgery stratified by histology (*N* = 366)

	Adenocarinoma	Nonadenocarcinoma	*P*
	*n* = 218	*n* = 148	
Age (median [range])	63.00 [31.00, 87.00]	64.00 [33.00, 88.00]	0.53
Male gender (%)	120 (55)	79 (53)	0.84
Number of brain metastases (%)			0.22
1	106 (49)	83 (56)	
2	45 (21)	34 (23)	
3	34 (16)	17 (12)	
4 +	33 (15)	14 (9.5)	
Systemic disease burden (%)			0.52
None	49 (23)	31 (21)	
Oligometastatic	107 (49)	80 (54)	
Unknown	4 (1.8)	5 (3.4)	
Widespread	58 (27)	32 (22)	
Systemic disease status (%)			0.40
Progressive	71 (33)	44 (30)	
Stable	132 (61)	88 (60)	
Unknown	15 (6.9)	16 (11)	
KPS (%)			0.30
50	1 (0.50)	0 (0.0)	
60	14 (6.4)	16 (11)	
70	34 (16)	18 (12)	
80	96 (44)	69 (47)	
90	69 (32)	39 (26)	
100	4 (1.8)	6 (4.1)	
DSGPA (%)			0.47
0	3 (1.4)	0 (0.0)	
0.5	9 (4.1)	10 (6.8)	
1	41 (19)	28 (19)	
1.5	65 (30)	41 (28)	
2	37 (17)	29 (20)	
2.5	34 (16)	23 (16)	
3	22 (10)	9 (6.1)	
3.5	6 (2.8)	8 (5.4)	
4	1 (0.50)	0 (0.0)	
Smoking Status (%)			0.40
Current	77 (36)	60 (41)	
Noncurrent	135 (62)	88 (59)	
Unknown	2 (0.90)	0 (0.0)	
Pack years (median [IQR])	40 [20, 50]	40 [20, 50]	0.60
Metastasis number (median [range])	2.00 [1.00, 13.00]	1.00 [1.00, 18.00]	0.074
Minimal_dose (median [range])	20.00 [11.00, 24.00]	20.00 [10.00, 24.00]	0.71

KPS, Karnofsky performance status; DS‐GPA, disease‐specific Graded Prognostic Assessment.

### Effect of current smoking status on clinical outcomes

**Figure 1:**
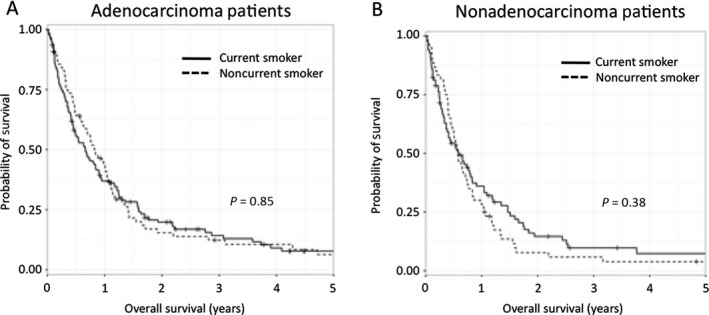
Kaplan–Meier curves for overall survival based on current smoking status. (A) Noncurrent smokers vs current smokers in the adenocarcinoma population and (B) Noncurrent smokers vs current smokers in the nonadenocarcinoma population.

The effect of current smoking status (current vs. not current) was evaluated for multiple clinical endpoints including survival, neurologic death and patterns of failure. On Cox Proportional Hazards analysis, current smoking status was not associated with overall survival for either adenocarcinoma (HR = 1.03, CI: 0.76–1.39, *P* = 0.85) or nonadenocarcinoma (HR = 0.85, CI: 0.60–1.21, *P* = 0.38) patients. Figure 1 shows the Kaplan–Meier plots of overall survival for patients based on smoking status.

**Figure 2:**
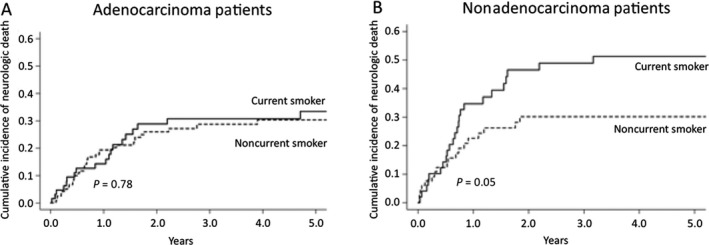
Cumulative incidence curves of neurologic death based on current smoking status. (A) Noncurrent smokers versus current smokers in the adenocarcinoma population and (B) Noncurrent smokers versus current smokers in the nonadenocarcinoma population.

The risk of neurologic death based on current smoking status was evaluated by competing risk analysis when patients were stratified by histologic subtype. The cumulative incidence of neurologic death at 6, 12, and 24 months was not affected in adenocarcinoma patients (13%, 14%, 29% for current smokers vs. 10%, 19%, and 26% for noncurrent smokers, *P* = 0.78). However, the cumulative incidence of neurologic death at 6, 12, and 24 months for nonadenocarcinoma demonstrated a trend toward being increased in current smokers compared to patients who had quit smoking for at least 1 year (14%, 35%, and 47% for current smokers vs. 12%, 23%, and 30% for noncurrent smokers, *P* = 0.053) (Fig. 2).

Local failure within SRS volume was evaluated by competing risk analysis. The cumulative incidence of local failure for the overall cohort at 6, 12, and 24 months was 1%, 4%, and 6%. The cumulative incidence of local failure at 6, 12, and 24 months for adenocarcinoma patients was 0%, 3%, 6% for current smokers and 2%, 4%, and 5% for noncurrent smokers (*P* = 0.95). The cumulative incidence of local failure at 6, 12, and 24 months for nonadenocarcinoma patients were 0%, 3%, 5% for current smokers and 2%, 5%, and 8% for noncurrent smokers (*P* = 0.77).

Regressive analyses revealed that noncurrent and current smokers had median brain metastasis velocities of 1.9 and 5.1 new metastases per year for patients with adenocarcinoma (*P* = 0.0013), and 2.8 and 2.1 new metastases per year for nonadenocarcinoma (*P* = 0.47). The effect of current smoking status on adenocarcinoma brain metastasis velocity remained on multiple regression when including potential confounding variables (*P* = 0.026).

### Effect of cumulative pack years on clinical outcomes

The effect of cumulative pack years smoking as a continuous variable was evaluated for multiple clinical endpoints including survival, neurologic death and patterns of failure. On Cox Proportional Hazards analysis, cumulative pack years smoking was not associated with overall survival for either adenocarcinoma (HR = 1.00, CI: 1.00–1.01, *P* = 0.18) or nonadenocarcinoma (HR = 1.01, CI: 1.00–1.01, *P* = 0.078) patients. On competing risk analysis, cumulative pack years smoking was not associated with the risk of neurologic death for adenocarcinoma (HR = 1.00, CI: 0.99–1.01, *P* = 0.56), but was associated with an increased risk of neurologic death for nonadenocarcinoma (HR = 1.01, CI: 1.00–1.02, *P* = 0.046).

Local failure within SRS volume was evaluated by competing risk analysis. The cumulative incidence of local failure at 6, 12, and 24 months for adenocarcinoma patients was 5%, 8%, 11% for <20 pack years, and 1%, 4%, and 5% for 20–40 pack years, and 0%, 2%, 5% for >40 pack years (*P* = 0.94). The cumulative incidence of local failure at 6, 12, and 24 months for nonadenocarcinoma patients was 9%, 14%, 24% for <20 pack years, and 0%, 4%, and 4% for 20–40 pack years, and 0%, 2%, 4% for >40 pack years (*P* = 0.039).

**Figure 3:**
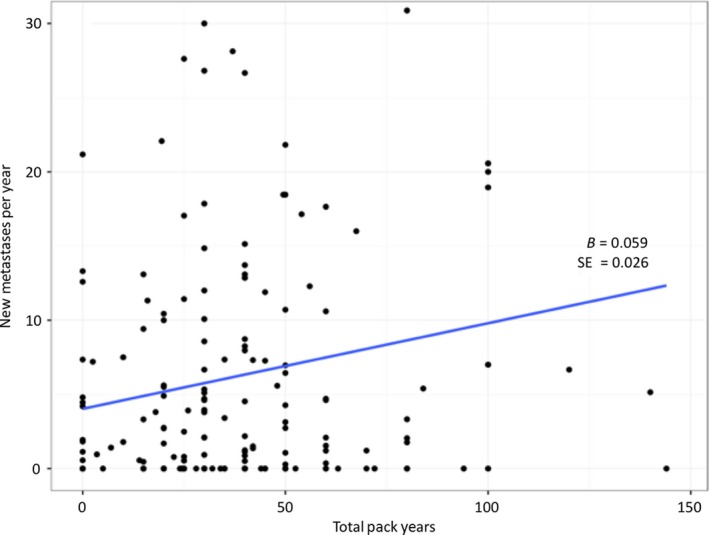
Brain metastasis velocity versus total pack year plots for lung cancer patients.

Regressive analyses revealed that patients with <20, 20–40, and >40 pack year history of smoking had median brain metastasis velocities of 1.9, 3.4 and 2.1 new metastases per year for adenocarcinoma (*P* = 0.34) and 1.4, 1.7, and 4.9 new metastases per year for nonadenocarcinoma (*P* = 0.034). The effect of cumulative pack years smoking on brain metastasis velocity was lost on multiple regression when including potential confounding variables (*P* = 0.20 for adenocarcinoma, *P* = 0.11 for nonadenocarcinoma). However, when all lung cancer patients were taken as a single population, cumulative pack years smoking showed a significant association with brain metastasis velocity on multiple regression when including potential confounding variables (*P* = 0.026) (Fig. 3). Table 2 shows the multiple regression model with the lowest AIC as determined by stepwise regression.

**Table 2:** Multivariate Model of brain metastasis velocity in lung cancer patients with brain metastases receiving intracranial stereotactic radiosurgery (*N* = 366)

Brain metastasis velocity^a^ (new metastases per year)									
	Adenocarcinoma (*n* = 76)			Nonadenocarcinoma (*n* = 106)			All patients (*n* = 182)^b^		
	*B*	CI	*P*	*B*	CI	*P*	*B*	CI	*P*
(Intercept)	15	3.0–27	0.015	4.2	−15–24	0.67	11	0.45–21	0.041
Age	−0.21	−0.37 to −0.05	0.013	−0.02	−0.30–0.27	0.9	−0.13	−0.28–0.01	0.075
DS‐GPA	−2.3	−6.0–1.5	0.24	−3.78	−10–2.7	0.25	−3.1	−6.4–0.26	0.07
Total pack years	0.040	−0.02–0.1	0.2	0.07	−0.020–0.16	0.11	0.06	0.01–0.11	0.024
Number of brain metastases at diagnosis	1.5	0.31–2.6	0.013	0.93	−0.68–2.54	0.25	1.2	0.27–2.1	0.012
Observations	106			76			182		
R^2^ /adj. R^2^	0.167/0.134			0.105/0.055			0.113/0.093		

DS‐GPA, disease‐specific Graded Prognostic Assessment.

a Brain metastasis velocity (BMV) was estimated by performing a separate linear regression versus time for each patient to obtain the slope representative of the best fit line. Clinical predictor variables of interest were included in putative multiple regression models for the outcome variable BMV. Stepwise regression methods were applied to determine the model with the lowest Akaike Information Criterion. *B * =  unstandardized regression coefficients. CI = 95% confidence interval.

b BMV was unable to be estimated for all patients due to censoring of some patients due to early death.

## Discussion

The current dataset showed an association between a greater cumulative pack year history of smoking with a greater brain metastasis velocity. The mechanism by which cumulative pack years affects brain metastasis velocity may be due to a greater exposure to carcinogens found in cigarette smoke, and how tobacco promotes a prosurvival and metastatic phenotype in cancers 1, 26, 27. A greater number of cells that reach a brain metastasis phenotype would potentially lead to more brain metastases. The observed decrease in local failure in adenocarcinoma patients with greater with greater cumulative pack year history may be due to a greater neuroendocrine differentiation of cancers more related to smoking as these cancers have also been considered to be radio‐responsive 28, 29.

Paradoxically, there was not an increase in either local failure or brain metastasis velocity in the nonadenocarcinoma patients who were active smokers, in spite of the strong trend toward greater neurologic death. Neurologic death can be caused by several factors including intracranial progression, leptomeningeal disease, toxicity of treatment, and cumulative neurologic effects of multiple medical comorbidities 30. We hypothesize that current smoking and greater smoking history may worsen global health status sufficiently to cause the increase in neurologic death seen in the nonadenocarcinoma population. Lung cancer patients have been found to have compromised cognitive status prior to the diagnosis with brain metastases 31, and continued smoking may affect their neurocognitive reserve further.

There are several limitations to this study. As a retrospective review, it is limited to hypothesis generation and subject to the limits of data that can be abstracted from medical records. Moreover, it is difficult to accurately determine smoking status of patients from the electronic medical record due to inconsistent documentation, high smoking relapse rates, and patient nondisclosure of smoking status 32, 33, 34. Patients were not managed with dedicated smoking cessation resources, limiting the ability to infer that structured smoking cessation has an effect on outcome.

The current report is the first to describe significant associations between smoking and clinical outcomes in lung cancer patients receiving SRS for brain metastases, corroborating prior studies that showed smoking status of lung cancer patients affects clinical outcomes 3, 9, 26, 35, 36. Unlike prior studies, the current findings were not restricted to nonmetastatic lung cancer patients. Our report supports the call for all cancer patients to be encouraged to quit smoking. Recent National Comprehensive Cancer Network guidelines on Smoking Cessation provide a framework for intervening with cancer patients who smoke using pharmacologic therapy and counseling 37. Future prospective studies with robust self‐report and biochemical validation of smoking status, and accompanying biomarker analyses could validate this report and elucidate mechanisms by which continued smoking contributes to worse outcomes of metastatic lung cancer.

## Conflicts of Interest

Authors report no financial disclosures or conflicts of interest.
